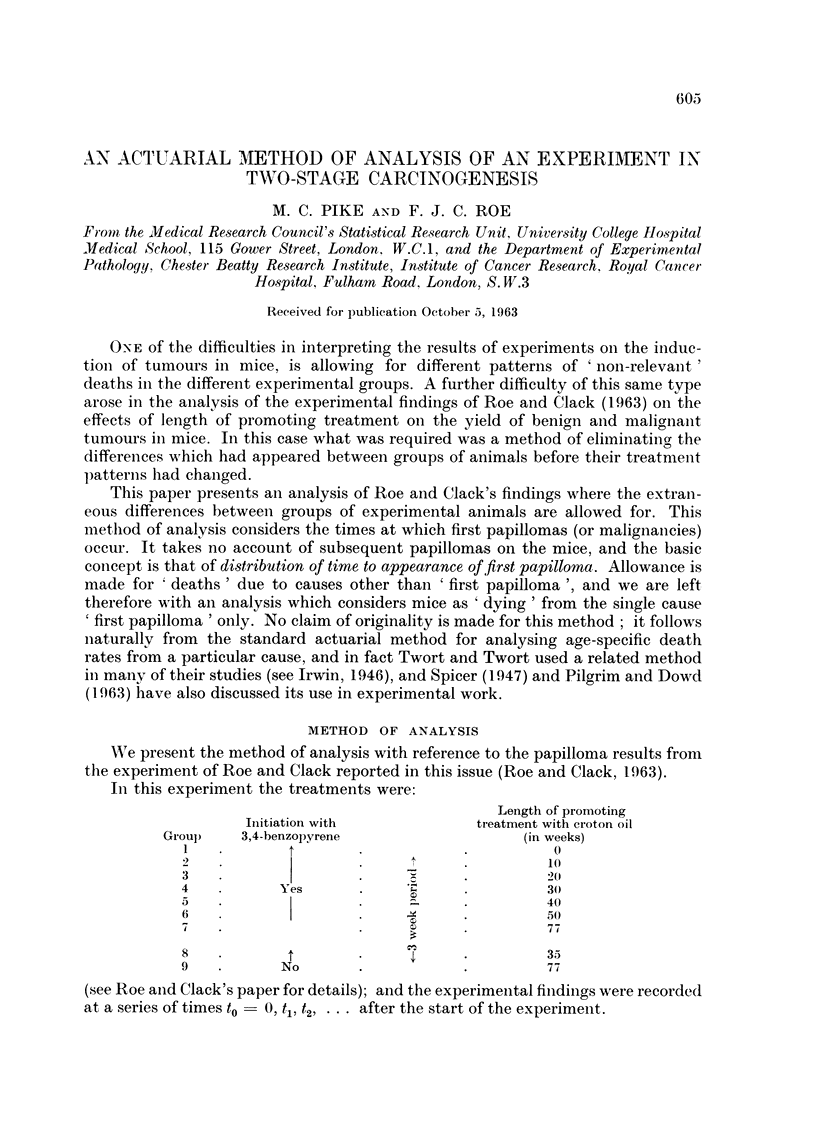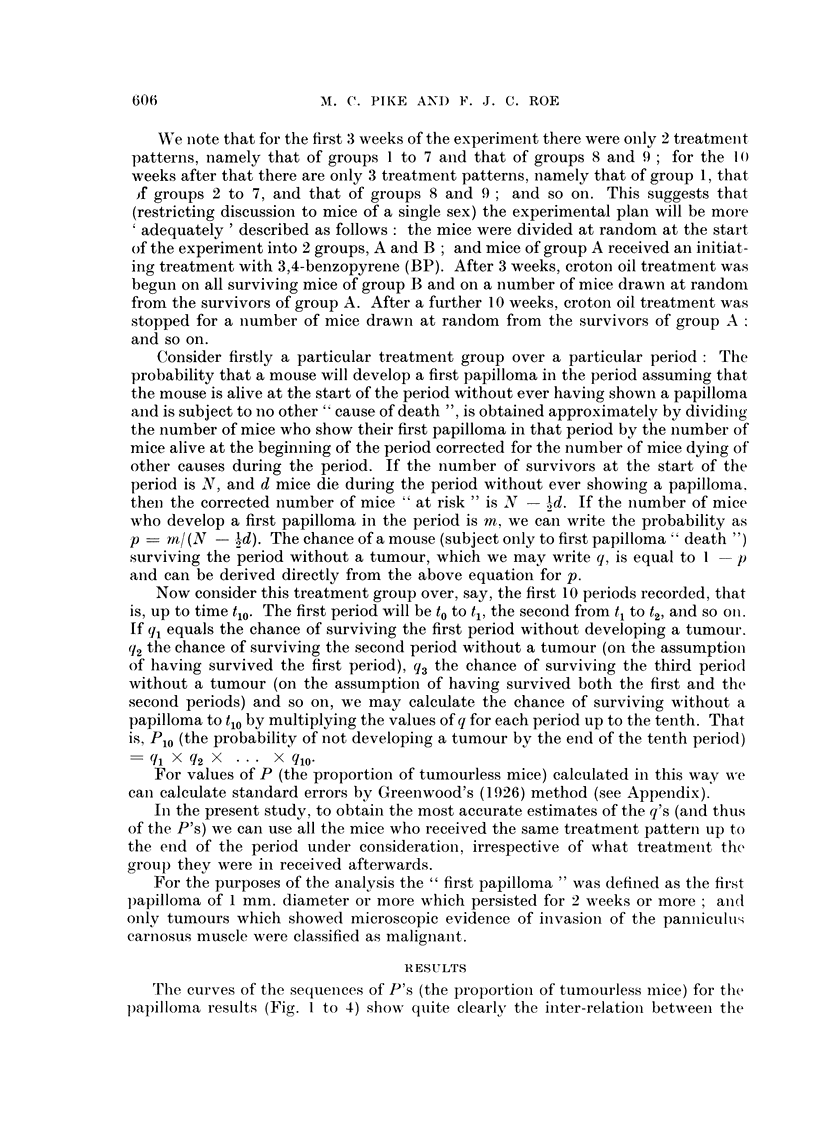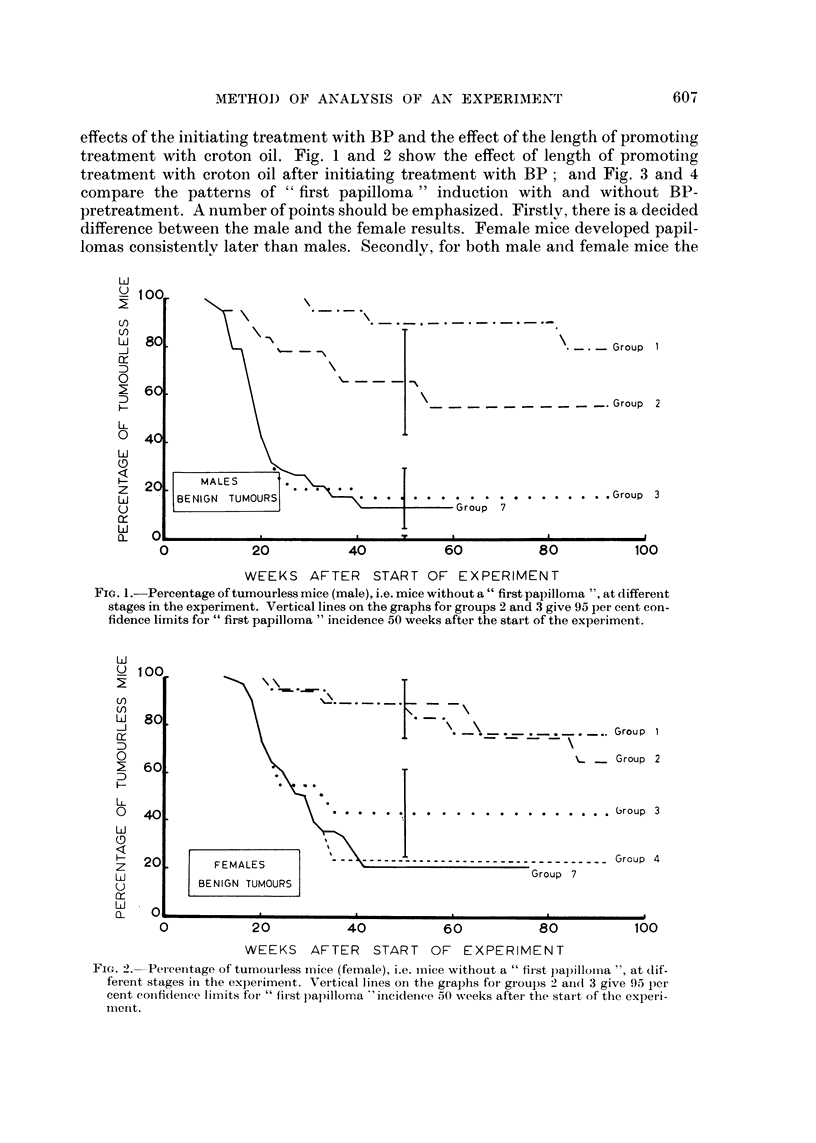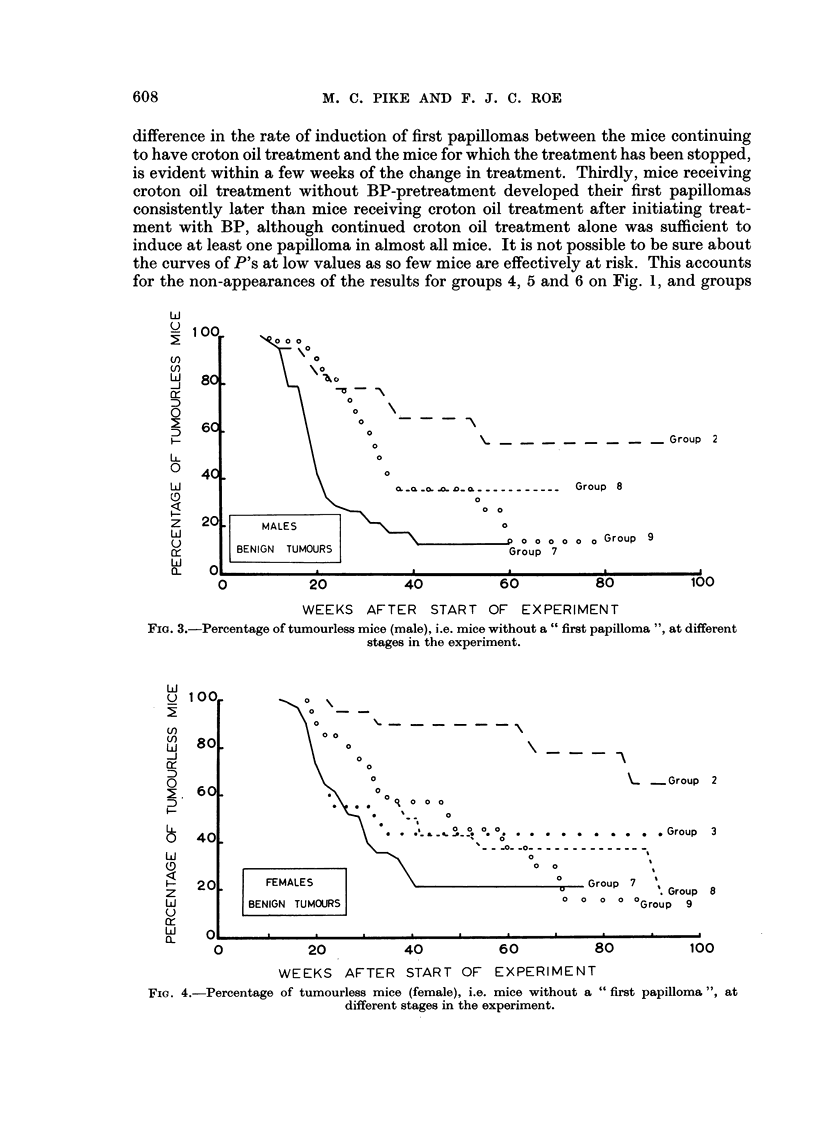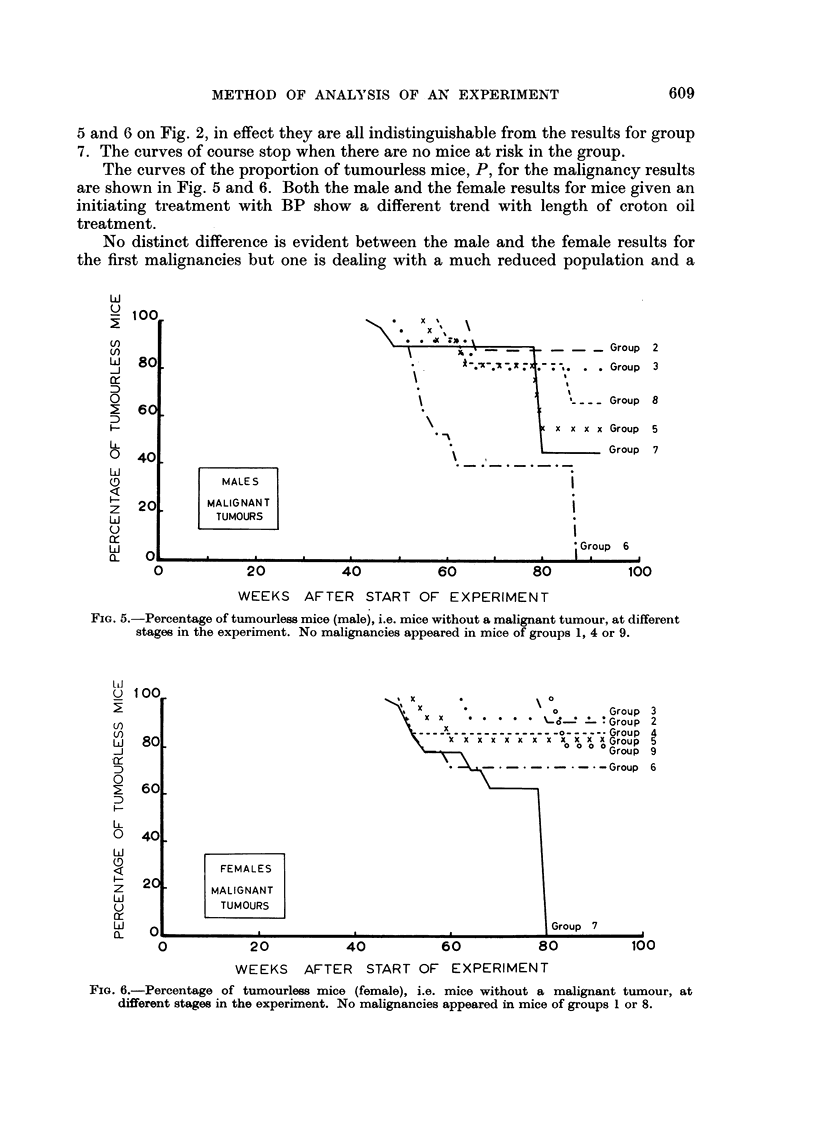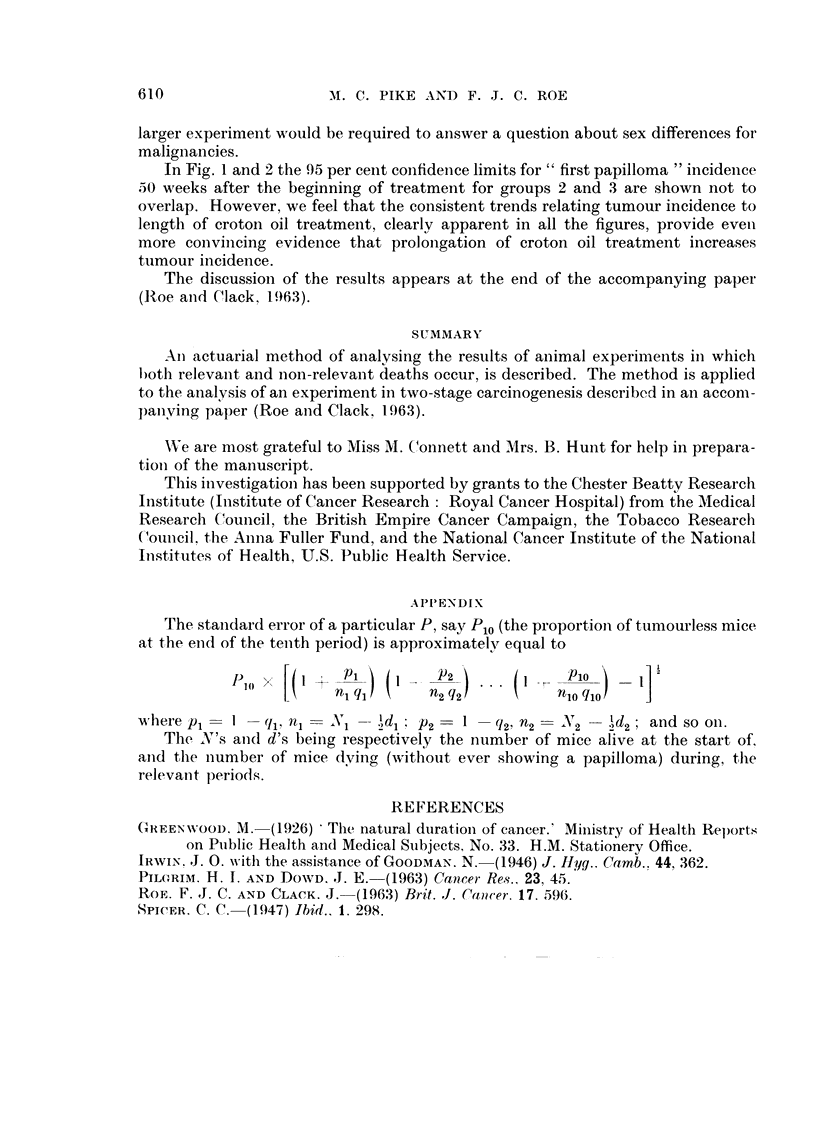# An Actuarial Method of Analysis of an Experiment in Two-Stage Carcinogenesis

**DOI:** 10.1038/bjc.1963.80

**Published:** 1963-12

**Authors:** M. C. Pike, F. J. C. Roe


### AN ACTUARIAL METHOD OF ANALYSIS OF AN EXPERIMENT IN TWO-STAGE CARCINOGENESIS

M. C. PIKE AND F. J. C. ROE

*From the Medical Research Council's Statistical Research Unit, University College Hospital Medical School,* 115 *Gower Street, London, W.C.*1, *and the Department of Experimental Pathology, Chester Beatty Research Institute, Institute of Cancer Research, Royal Cancer Hospital, Fulham Road, London, S.W.*3

Received for publication October 5, 1963

ONE of the difficulties in interpreting the results of experiments on the induction of tumours in mice, is allowing for different patterns of ' non-relevant ' deaths in the different experimental groups. A further difficulty of this same type arose in the analysis of the experimental findings of Roe and Clack (1963) on the effects of length of promoting treatment on the yield of benign and malignant tumours in mice. In this case what was required was a method of eliminating the differences which had appeared between groups of animals before their treatment patterns had changed.

This paper presents an analysis of Roe and Clack's findings where the extraneous differences between groups of experimental animals are allowed for. This method of analysis considers the times at which first papillomas (or malignancies) occur. It takes no account of subsequent papillomas on the mice, and the basic concept is that of *distribution of time to appearance of first papilloma.* Allowance is made for ' deaths ' due to causes other than ' first papilloma ', and we are left therefore with an analysis which considers mice as ' dying ' from the single cause ' first papilloma ' only. No claim of originality is made for this method ; it follows naturally from the standard actuarial method for analysing age-specific death rates from a particular cause, and in fact Twort and Twort used a related method in many of their studies (see Irwin, 1946), and Spicer (1947) and Pilgrim and Dowd (1963) have also discussed its use in experimental work.

### METHOD OF ANALYSIS

We present the method of analysis with reference to the papilloma results from the experiment of Roe and Clack reported in this issue (Roe and Clack, 1963).

In this experiment the treatments were:

| Group | Initiation with 3,4-benzopyrene | | Length of promoting treatment with croton oil (in weeks) |
|---|---|---|---|
| 1 | ↑ | | 0 |
| 2 | | ↑ | 10 |
| 3 | | | 20 |
| 4 | Yes | | 30 |
| 5 | | ←3 week period→ | 40 |
| 6 | | | 50 |
| 7 | | | 77 |
| 8 | ↑ | ↓ | 35 |
| 9 | No | | 77 |

(see Roe and Clack's paper for details); and the experimental findings were recorded at a series of times $t_0 = 0, t_1, t_2, \ldots$ after the start of the experiment.

We note that for the first 3 weeks of the experiment there were only 2 treatment patterns, namely that of groups 1 to 7 and that of groups 8 and 9 ; for the 10 weeks after that there are only 3 treatment patterns, namely that of group 1, that of groups 2 to 7, and that of groups 8 and 9 ; and so on. This suggests that (restricting discussion to mice of a single sex) the experimental plan will be more ' adequately ' described as follows : the mice were divided at random at the start of the experiment into 2 groups, A and B ; and mice of group A received an initiating treatment with 3,4-benzopyrene (BP). After 3 weeks, croton oil treatment was begun on all surviving mice of group B and on a number of mice drawn at random from the survivors of group A. After a further 10 weeks, croton oil treatment was stopped for a number of mice drawn at random from the survivors of group A ; and so on.

Consider firstly a particular treatment group over a particular period : The probability that a mouse will develop a first papilloma in the period assuming that the mouse is alive at the start of the period without ever having shown a papilloma and is subject to no other " cause of death ", is obtained approximately by dividing the number of mice who show their first papilloma in that period by the number of mice alive at the beginning of the period corrected for the number of mice dying of other causes during the period. If the number of survivors at the start of the period is $N$, and $d$ mice die during the period without ever showing a papilloma, then the corrected number of mice " at risk " is $N - \frac{1}{2}d$. If the number of mice who develop a first papilloma in the period is $m$, we can write the probability as $p = m/(N - \frac{1}{2}d)$. The chance of a mouse (subject only to first papilloma " death ") surviving the period without a tumour, which we may write $q$, is equal to $1 - p$ and can be derived directly from the above equation for $p$.

Now consider this treatment group over, say, the first 10 periods recorded, that is, up to time $t_{10}$. The first period will be $t_0$ to $t_1$, the second from $t_1$ to $t_2$, and so on. If $q_1$ equals the chance of surviving the first period without developing a tumour, $q_2$ the chance of surviving the second period without a tumour (on the assumption of having survived the first period), $q_3$ the chance of surviving the third period without a tumour (on the assumption of having survived both the first and the second periods) and so on, we may calculate the chance of surviving without a papilloma to $t_{10}$ by multiplying the values of $q$ for each period up to the tenth. That is, $P_{10}$ (the probability of not developing a tumour by the end of the tenth period) $= q_1 \times q_2 \times \ldots \times q_{10}$.

For values of $P$ (the proportion of tumourless mice) calculated in this way we can calculate standard errors by Greenwood's (1926) method (see Appendix).

In the present study, to obtain the most accurate estimates of the $q$'s (and thus of the $P$'s) we can use all the mice who received the same treatment pattern up to the end of the period under consideration, irrespective of what treatment the group they were in received afterwards.

For the purposes of the analysis the " first papilloma " was defined as the first papilloma of 1 mm. diameter or more which persisted for 2 weeks or more ; and only tumours which showed microscopic evidence of invasion of the panniculus carnosus muscle were classified as malignant.

RESULTS

The curves of the sequences of $P$'s (the proportion of tumourless mice) for the papilloma results (Fig. 1 to 4) show quite clearly the inter-relation between the effects of the initiating treatment with BP and the effect of the length of promoting treatment with croton oil. Fig. 1 and 2 show the effect of length of promoting treatment with croton oil after initiating treatment with BP ; and Fig. 3 and 4 compare the patterns of " first papilloma " induction with and without BP-pretreatment. A number of points should be emphasized. Firstly, there is a decided difference between the male and the female results. Female mice developed papillomas consistently later than males. Secondly, for both male and female mice the

FIG. 1.—Percentage of tumourless mice (male), i.e. mice without a " first papilloma ", at different stages in the experiment. Vertical lines on the graphs for groups 2 and 3 give 95 per cent confidence limits for " first papilloma " incidence 50 weeks after the start of the experiment.

FIG. 2.—Percentage of tumourless mice (female), i.e. mice without a " first papilloma ", at different stages in the experiment. Vertical lines on the graphs for groups 2 and 3 give 95 per cent confidence limits for " first papilloma " incidence 50 weeks after the start of the experiment.

difference in the rate of induction of first papillomas between the mice continuing to have croton oil treatment and the mice for which the treatment has been stopped, is evident within a few weeks of the change in treatment. Thirdly, mice receiving croton oil treatment without BP-pretreatment developed their first papillomas consistently later than mice receiving croton oil treatment after initiating treatment with BP, although continued croton oil treatment alone was sufficient to induce at least one papilloma in almost all mice. It is not possible to be sure about the curves of $P$'s at low values as so few mice are effectively at risk. This accounts for the non-appearances of the results for groups 4, 5 and 6 on Fig. 1, and groups

Fig. 3.—Percentage of tumourless mice (male), i.e. mice without a " first papilloma ", at different stages in the experiment.

Fig. 4.—Percentage of tumourless mice (female), i.e. mice without a " first papilloma ", at different stages in the experiment.

5 and 6 on Fig. 2, in effect they are all indistinguishable from the results for group 7. The curves of course stop when there are no mice at risk in the group.

The curves of the proportion of tumourless mice, $P$, for the malignancy results are shown in Fig. 5 and 6. Both the male and the female results for mice given an initiating treatment with BP show a different trend with length of croton oil treatment.

No distinct difference is evident between the male and the female results for the first malignancies but one is dealing with a much reduced population and a

FIG. 5.—Percentage of tumourless mice (male), i.e. mice without a malignant tumour, at different stages in the experiment. No malignancies appeared in mice of groups 1, 4 or 9.

FIG. 6.—Percentage of tumourless mice (female), i.e. mice without a malignant tumour, at different stages in the experiment. No malignancies appeared in mice of groups 1 or 8.

larger experiment would be required to answer a question about sex differences for malignancies.

In Fig. 1 and 2 the 95 per cent confidence limits for " first papilloma " incidence 50 weeks after the beginning of treatment for groups 2 and 3 are shown not to overlap. However, we feel that the consistent trends relating tumour incidence to length of croton oil treatment, clearly apparent in all the figures, provide even more convincing evidence that prolongation of croton oil treatment increases tumour incidence.

The discussion of the results appears at the end of the accompanying paper (Roe and Clack, 1963).

SUMMARY

An actuarial method of analysing the results of animal experiments in which both relevant and non-relevant deaths occur, is described. The method is applied to the analysis of an experiment in two-stage carcinogenesis described in an accompanying paper (Roe and Clack, 1963).

We are most grateful to Miss M. Connett and Mrs. B. Hunt for help in preparation of the manuscript.

This investigation has been supported by grants to the Chester Beatty Research Institute (Institute of Cancer Research : Royal Cancer Hospital) from the Medical Research Council, the British Empire Cancer Campaign, the Tobacco Research Council, the Anna Fuller Fund, and the National Cancer Institute of the National Institutes of Health, U.S. Public Health Service.

APPENDIX

The standard error of a particular $P$, say $P_{10}$ (the proportion of tumourless mice at the end of the tenth period) is approximately equal to

$$P_{10} \times \left[\left(1+\frac{p_1}{n_1 q_1}\right)\left(1+\frac{p_2}{n_2 q_2}\right)\ldots\left(1+\frac{p_{10}}{n_{10} q_{10}}\right)-1\right]^{\frac{1}{2}}$$

where $p_1 = 1 - q_1$, $n_1 = N_1 - \frac{1}{2}d_1$ ; $p_2 = 1 - q_2$, $n_2 = N_2 - \frac{1}{2}d_2$ ; and so on.

The $N$'s and $d$'s being respectively the number of mice alive at the start of, and the number of mice dying (without ever showing a papilloma) during, the relevant periods.

REFERENCES

GREENWOOD, M.—(1926) 'The natural duration of cancer.' Ministry of Health Reports on Public Health and Medical Subjects, No. 33. H.M. Stationery Office.
IRWIN, J. O. with the assistance of GOODMAN, N.—(1946) *J. Hyg., Camb.*, **44**, 362.
PILGRIM, H. I. AND DOWD, J. E.—(1963) *Cancer Res.*, **23**, 45.
ROE, F. J. C. AND CLACK, J.—(1963) *Brit. J. Cancer*, **17**, 596.
SPICER, C. C.—(1947) *Ibid.*, **1**, 298.